# Supplementary material for: Diversity and Biotechnological Potential of Cultivable Halophilic and Halotolerant Bacteria from the “Los Negritos” Geothermal Area
Source: Microorganisms. 2024 Feb 27;12(3):482. doi: 10.3390/microorganisms12030482 (PMC10972316; doi:10.3390/microorganisms12030482)
Supplement: Supplementary file 1 [file microorganisms-12-00482-s001.zip › Suplementary_figures.pdf]

Suplementary Material

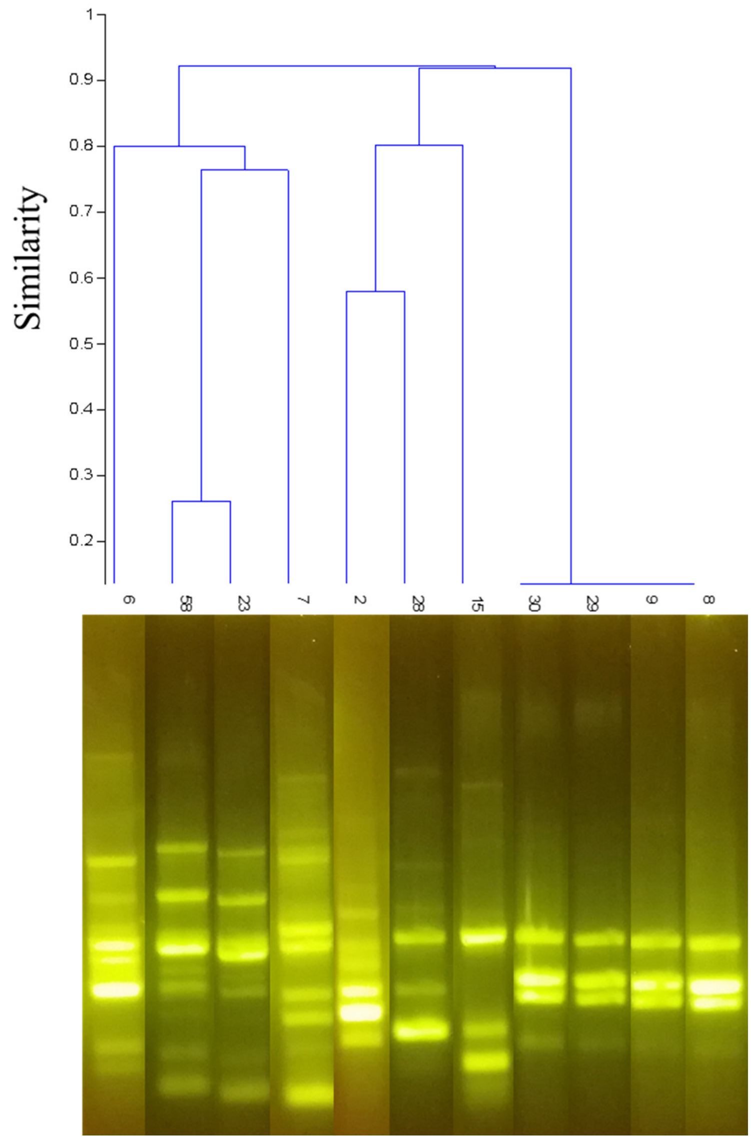

Figure S1. Band pattern obtained by BOX-PCR of the strains of the genus *Halomonas* isolated from saline agricultural soils of “Los Negritos” - Villamar - Michoacán. The patterns obtained grouped the strains into six groups, where the following strains were found: 6) *Halomonas* sp. LNSP6E3-2; 23, 58) *Halomonas* sp. LNSP10E3-2.1 and LNSP3103-1; 7) *Halomonas* sp. LNSP6-1; 2, 28) *Halomonas* sp. LNSP2E3-1 and LNSP4103-1; 15) *Halomonas* sp. LNSP4E3-1; 8, 9, 29, 30) *Halomonas* sp. LNSP5E3-1, LNSP5E3-2, LNSP5E3-1.1 and LNSP5E3-2.2.

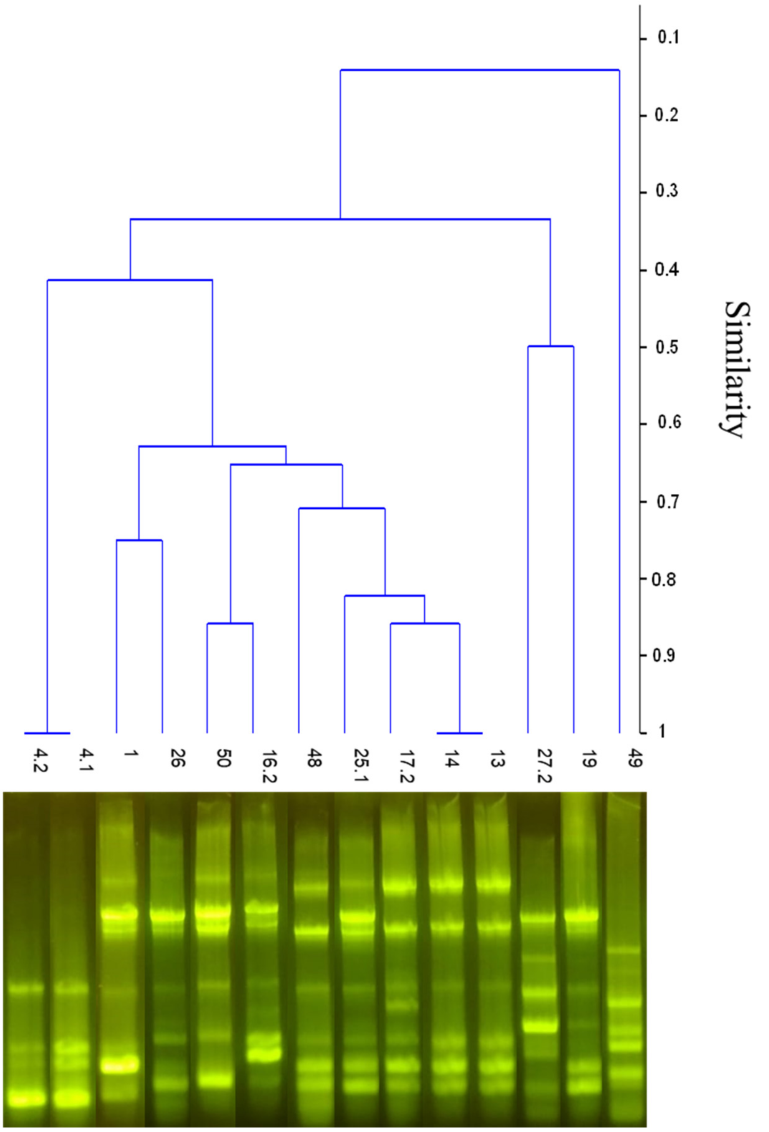

Figure S2. Band patterns obtained by BOX-PCR of the *Oceanobacillus* strains isolated from saline agricultural soils of "Los Negritos" - Villamar - Michoacán. Eight patterns were identified, where the following strains were found: 4.1, 4.2) *Oceanobacillus* sp. LNSP2103-1.1 and LNSP2103-1.2; 1, 26) *Oceanobacillus* sp. LNSP2E3-2, LNSP1E3-1.1; 16.2, 50) *Oceanobacillus* sp. LNSP2E3-1.2, LNSP3E3-2; 48) *Oceanobacillus* sp. LNSP10E3-2; 25.1) *Oceanobacillus* sp. LNSP9E3-2.1; 17.2) *Oceanobacillus* sp. LNSP7E3-1.2; 13, 14) *Oceanobacillus* sp. LNSP8E3-1, LNSP8E3-2; 19, 27.2) *Oceanobacillus* sp. LNSP1E3-1, LNSP9E3-1.2, and 49) *Oceanobacillus* sp. LNSP3E3-1.

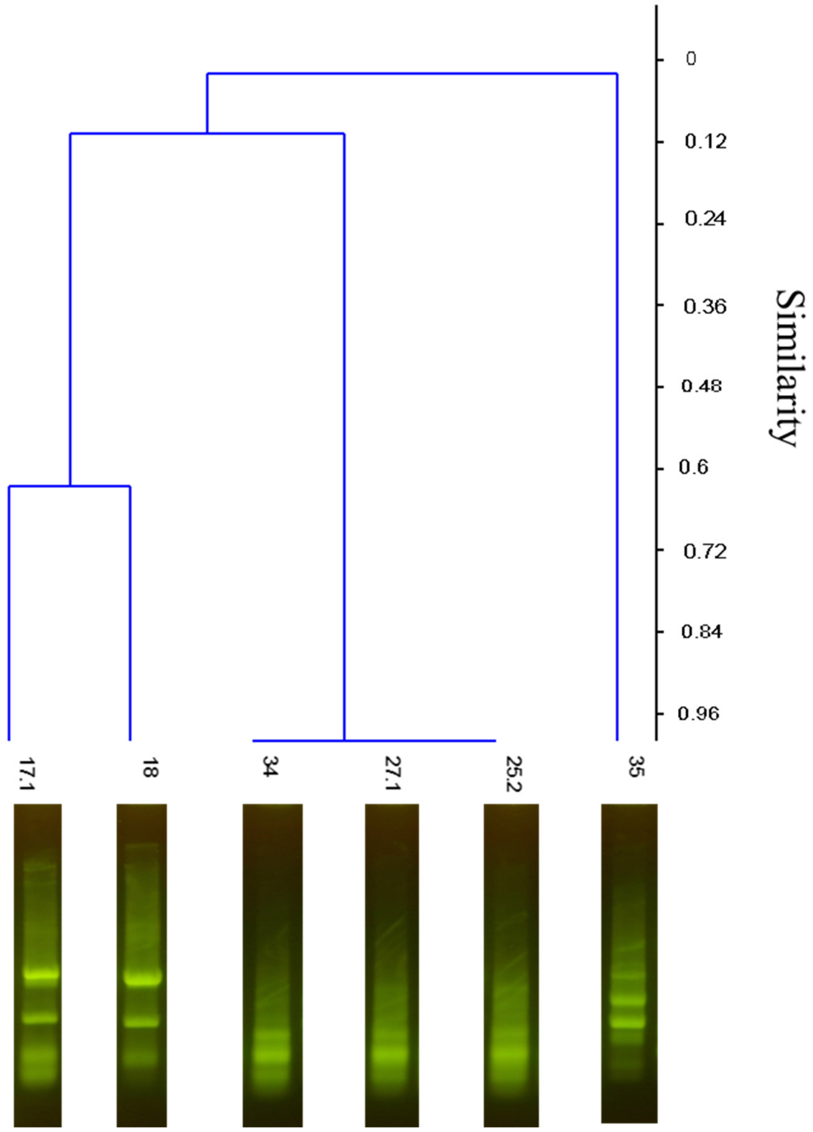

Figure S3. Band pattern obtained by BOX-PCR of *Staphylococcus* strains isolated from saline agricultural soils of "Los Negritos" - Villamar - Michoacán. Three patterns were identified, where the following strains were found: 17.1, 18) *Staphylococcus* sp. LNSP7E3-1.1, LNSP7E3-2; 25.2, 27.1, 34) *Staphylococcus* sp. LNSP9E3-2.2, LNSP9E3-1.1, LNSP4105-1; 35) *Staphylococcus* sp. LNSP9105-1.

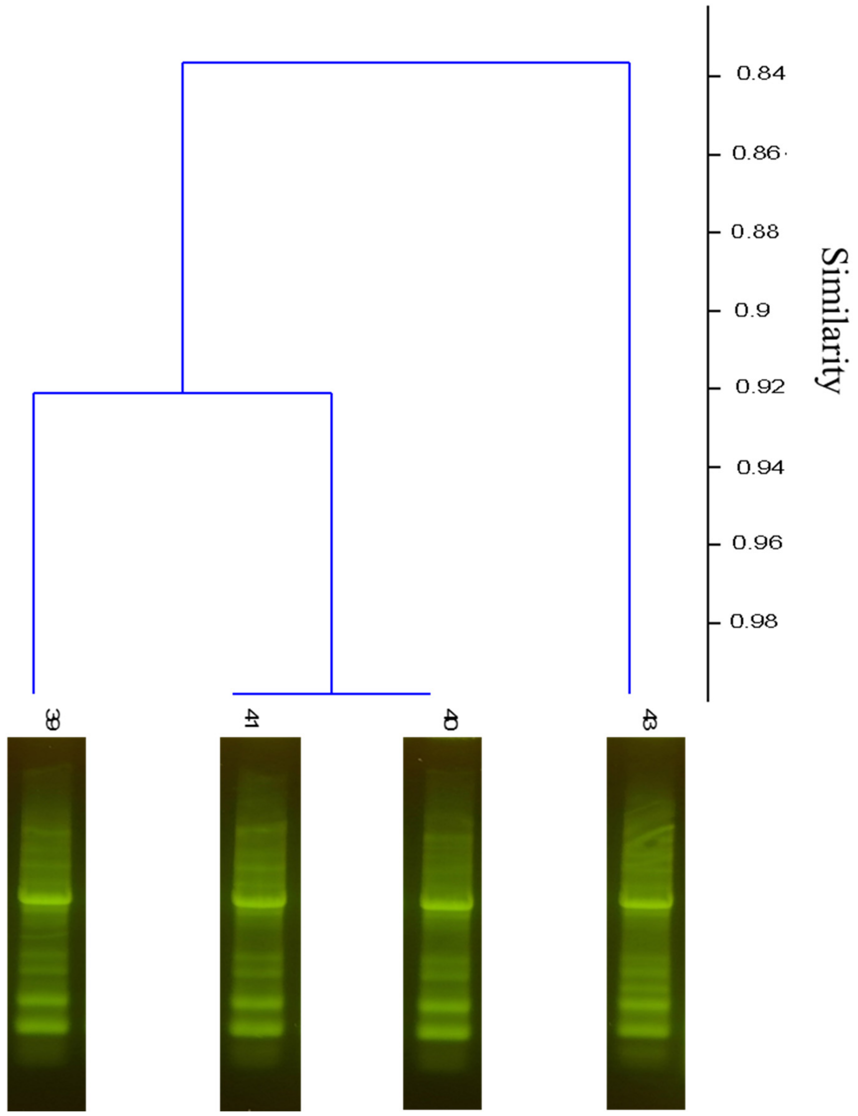

Figure S4. Band pattern obtained by BOX-PCR of *Salimicrobium* strains isolated from saline agricultural soils of "Los Negritos" - Villamar - Michoacán. Three patterns were identified, where the following strains were found: 39) *Salimicrobium* sp. LNHM3E3-1.1; 40, 41) *Salimicrobium* sp. LNHM3E3-1, LNHM2E3-1; 43) *Salimicrobium* sp. LNHM10E3-1.

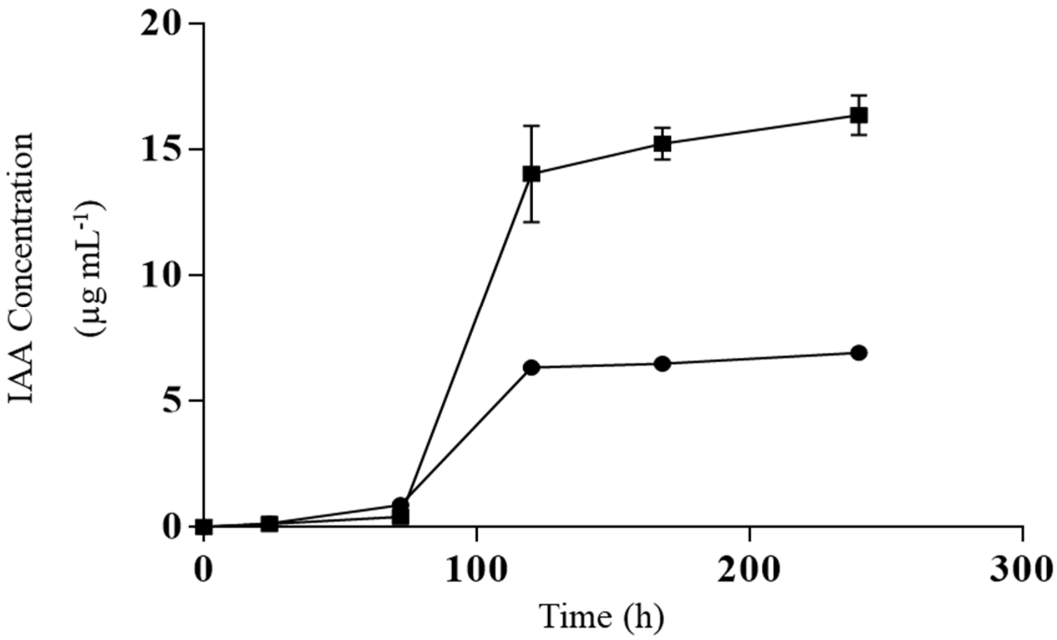

Figure S5. Production of IAA by *Salibacterium* sp. LNHM5E3-2.2 strain in two different media supplemented with 10% NaCl. HM medium supplemented with L-tryptophan (■); JP medium (●).

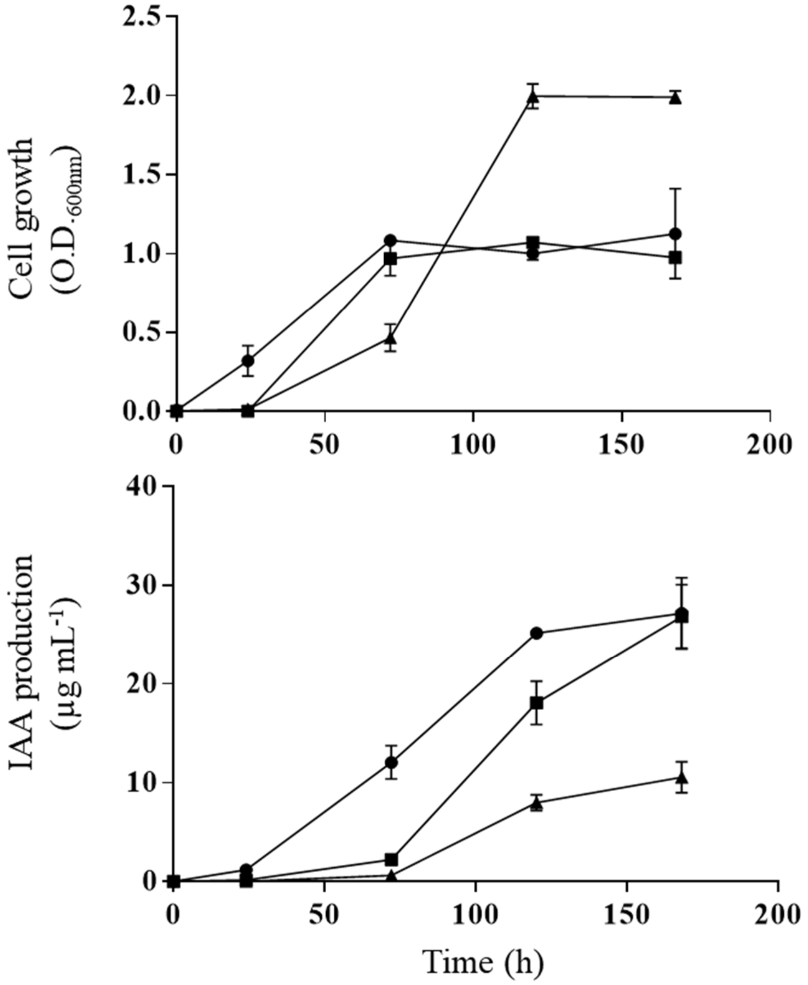

Figure S6. Kinetics of cell growth and IAA production by the strains *A. brasilense* and *Salibacterium* sp. LNHM5E3-2.2 in JP medium with and without salinity stress. a) Growth and b) IAA production by *A. brasilense* 0% NaCl (●); *Salibacterium* sp. LNHM5E3-2.2 0% NaCl (■); *Salibacterium* sp. LNHM5E3-2.2 10% NaCl (▲).
