# Supplementary material for: Diversity and Biotechnological Potential of Cultivable Halophilic and Halotolerant Bacteria from the “Los Negritos” Geothermal Area
Source: Microorganisms. 2024 Feb 27;12(3):482. doi: 10.3390/microorganisms12030482 (PMC10972316; doi:10.3390/microorganisms12030482)
Supplement: Supplementary file 1 [file microorganisms-12-00482-s001.zip › Table-S1.pdf]

Table S1. Halophilic and halotolerant bacterial diversity isolated from arable and saline soils from “Los Negritos” – Villamar-Michoacán.

Site	CFU gs^{-1} [a]		Strain	Microscopic morphology [b]	NaCl tolerance (%)
	TSA	SP 10% NaCl			
AS1	2.6x10^{7}	5.5x10^{5}	*Bacillus* sp. LNSP2103-3	G + rods	0-20
			Bacillus sp. LNSP2103-4		
			Halomonas sp. LNSP2E3-1	G – rods	0.20
			Halomonas sp. LNSP3103-1		0-12.5
			Halomonas sp. LNSP10E3-2.1		0-20
			Marinococcus sp. LNHM3E3-2.2	G + cocci	0-25
			Oceanobacillus sp. LNSP2103-1.1	G + rods	0-20
			Oceanobacillus sp. LNSP2103-1.2		
			Oceanobacillus sp. LNSP2E3-1.2		
			Oceanobacillus sp. LNSP1E3-1		
			Oceanobacillus sp. LNSP1E3-1.1		0-17.5
			Oceanobacillus sp. LNSP3E3-1		0-20
			Oceanobacillus sp. LNSP3E3-2		
			Oceanobacillus sp. LNSP10E3-2_0		5-17.5
			Oceanobacillus sp. LNSP2E3-2		5-20
			Salimicrobium sp. LNHM10E3-1		5-22
			Salimicrobium sp. LNHM2E3-1		5-22
			Salimicrobium sp. LNHM3E3-1.1		5-22
			Salimicrobium sp. LNHM3E3-1		0.22
			Salinicoccus sp. LNSP10E3-1.1	G + cocci	0-15
			Terribacillus sp. LNSP3105-1	G + rods	0-12.5
			Virgibacillus sp. LNSP10E3-1		0-20
			Virgibacillus sp. LNSP2E3-1.1		
S	2.1x10^{7}	5x10^{5}	*Brachybacterium* sp. LNSP6-4	G + cocci	0-12.5
			Gracilibacillus sp. LNSP5103-2	G + rods	0-20
			Halobacillus sp. LNHM4103-1		0.22
			Halobacillus sp. LNHM5103-1		
			Halobacillus sp. LNSP6-3.1		2.5-10
			Halomonas sp. LNSP4103-1	G - rods	2.5-15
			Halomonas sp. LNSP4E3-1		0-20
			Halomonas sp. LNSP5E3-1		2.5-20

			Halomonas sp. LNSP5E3-2		
			Halomonas sp. LNSP5E3-1.1		2.5-15
			Halomonas sp. LNSP5E3-2.2		2.5-20
			Halomonas sp. LNSP6-1		2.5-17.5
			Halomonas sp. LNSP6E3-2		0-17.5
			Kocuria sp. LNSP5103-1		0-10
			Marinococcus sp. LNHM5E3-2.1		0-25
			Marinococcus sp. LNSP5103-1.1	G + cocci	0-10
			Marinococcus sp. LNHM4E3-1		0-25
			Planococcus sp. LNSP5103-1.2		0-10
			Priestia sp. LNSP6-2		0-17.5
			Salibacterium sp. LNHM5E3-1	G + rods	2.5-25
			Salibacterium sp. LNHM5E3-2.2		5-25
			Salinicoccus sp. LNSP6E3-1	G + cocci	2.5-17.5
			Staphylococcus sp. LNSP4105-1		0-17.5
			Brachybacterium sp. LNSP7105-1.1		0-17.5
			Brachybacterium sp. LNSP9103-1.1	G + cocci	0-10
			Marinococcus sp. LNSP9103-4		2.5-20
			Nesterenkonia sp. LNSP9103-1		0-15
			Oceanobacillus sp. LNSP7E3-1.2		
			Oceanobacillus sp. LNSP8E3-1		0-20
			Oceanobacillus sp. LNSP8E3-2	G + rods	
AS2	4.4×10^7	6×10^5	*Oceanobacillus* sp. LNSP9E3-2.1		
			Oceanobacillus sp. LNSP9E3-1.2		2.5-20
			Planococcus sp. LNSP7105-1.2		0-17.5
			Staphylococcus sp. LNSP7E3-1.1		
			Staphylococcus sp. LNSP7E3-2	G + cocci	0-25
			Staphylococcus sp. LNSP9E3-2.2		
			Staphylococcus sp. LNSP9E3-1.1		
			Staphylococcus sp. LNSP9105-1		0-17.5
			Terribacillus sp. LNSP9103-2.2	G + rods	0-10

[a] Colony Forming Units per soil gram.

[b] Gram stain microscopic morphology.
