# Supplementary material for: Diversity and Biotechnological Potential of Cultivable Halophilic and Halotolerant Bacteria from the “Los Negritos” Geothermal Area
Source: Microorganisms. 2024 Feb 27;12(3):482. doi: 10.3390/microorganisms12030482 (PMC10972316; doi:10.3390/microorganisms12030482)
Supplement: Supplementary file 1 [file microorganisms-12-00482-s001.zip › Table-S2.pdf]

Table S2. Similarity among the isolated strains from arable and saline soils from "Los Negritos" - Villamar - Michoacán state compared with the type strains, close reference strain and the strains of this study (when apply).

Strain/Genera	Type species	Similarity with type strain (%)	Close related reference strain (%S) [a]	Similarity between strains (%)
		Terribacillus		
Terribacillus sp. LNSP9103-2.2	*T. saccharophilus*	96.1- 98		
Terribacillus sp. LNSP3105-1	002-048^{T}			96.3
		Staphylococcus		
Staphylococcus sp. LNSP7E3-1.1			97.8	
Staphylococcus sp. LNSP7E3-2			98	98.2-98.8
Staphylococcus sp. LNSP9E3-2.2	*S. aureus* subsp.		98.1	
Staphylococcus sp. LNSP9E3-1.1	*aureus*	95.5-96.3	97.5	
Staphylococcus sp. LNSP4105-1	DSM 20231^{T}		97.8	98
Staphylococcus sp. LNSP9105-1			98.1	
		Virgibacillus		
Virgibacillus sp. LNSP2E3-1.1	*V. pantothenticus*	94.5	97.6	97.6
Virgibacillus sp. LNSP10E3-1	DSM 26^{T}	94.6	97.8	
		Salibacterium		
Salibacterium sp. LNHM5E3-1	*S. halotolerans*	91.9	93	
Salibacterium sp. LNHM5E3-2.2	S7^{T}	92.5		96.8
		Salimicrobium		
Salimicrobium sp. LNHM3E3-1.1				
Salimicrobium sp. LNHM3E3-1	*S. album*	95.2-95.8	96.5-96.9	98.2-98.9
Salimicrobium sp. LNHM2E3-1	DSM 20748^{T}			
Salimicrobium sp. LNHM10E3-1				
		Priestia		
Priestia sp. LNSP6-2	*P. megaterium* NBRC 15308^{T}	90.3	96	NA
		Planococcus		
Planococcus sp. LNSP5103-1.2	*P. citreus*	97.1		98.3
Planococcus sp. LNSP7105-1.2	DSM 20549^{T}	96.9		
		Salinicoccus		
Salinicoccus sp. LNSP6E3-1	*S. roseus*	92.4	93.9	92
Salinicoccus sp. LNSP10E3-1.1	DSM 5351^{T}	96.8		
		Nesterenkonia		
Nesterenkonia sp. LNSP9103-1	*N. halobia* DSM 20541^{T}	95	97	NA
		Kocuria		
Kocuria sp. LNSP5103-1	*K. rosea* DSM 20447^{T}	96.1	97.4	NA
		Marinococcus		
Marinococcus sp. LNHM4E3-1			97.5	
Marinococcus sp. LNHM3E3-2.2	*M. halophilus*		97.4	
Marinococcus sp. LNHM5E3-2.1	KCTC 2843^{T}	96.7-97.6	96.7	96.9-98.7
Marinococcus sp. LNSP9103-4			97.1	
Marinococcus sp. LNSP5103-1.1			97.6	

Table S2. Continued

Strain/Genera	Type species	Similarity with type strain (%)	Close related reference strain (%S) [a]	Similarity between strains (%)
		Halomonas		
Halomonas sp. LNSP2E3-1	*H. elongata* DSM 2581^T	90.2-94.5	96	90.6-98
Halomonas sp. LNSP6E3-2			97	
Halomonas sp. LNSP6-1			96.1	
Halomonas sp. LNSP5E3-1			95.6	
Halomonas sp. LNSP5E3-2			96.6	
Halomonas sp. LNSP4E3-1			97.7	
Halomonas sp. LNSP10E3-2.1			97	
Halomonas sp. LNSP4103-1			96.7	
Halomonas sp. LNSP5E3-1.1			95.1	
Halomonas sp. LNSP5E3-2.2			95.2	
Halomonas sp. LNSP3103-1			97.3	
		Gracilibacillus		
Gracilibacillus sp. LNSP5103-2	*G. halotolerans* NN^T	94.1	97.1	NA
		Bacillus		
Bacillus sp. LNSP2103-3	*B. subtilis* NCIB 3610^T	97.8-98	98.4	98.5
Bacillus sp. LNSP2103-4			98.2	
		Brachybacterium		
Brachybacterium sp. LNSP6-4	*B. faecium* DSM 4810^T	96-96.3	96.7	96.2-98.5
Brachybacterium sp. LNSP9103-1.1			96.9	
Brachybacterium sp. LNSP7105-1.1			97.3	
		Halobacillus		
Halobacillus sp. LNSP6-3.1	*H. halophilus* DSM 2266^T	95.1-95.9	95.6	94.9-97.9
Halobacillus sp. LNHM5103-1			95.4	
Halobacillus sp. LNHM4103-1			97.4	
		Oceanobacillus		
Oceanobacillus sp. LNSP2E3-2	*O. iheyensis* HTE831^T	93.3-96.2	97.6	93-98.8
Oceanobacillus sp. LNSP2103-1.1			97.3	
Oceanobacillus sp. LNSP2103-1.2			95.9	
Oceanobacillus sp. LNSP8E3-1			97.8	
Oceanobacillus sp. LNSP8E3-2			96.9	
Oceanobacillus sp. LNSP2E3-1.2			97.3	
Oceanobacillus sp. LNSP7E3-1.2			96.6	
Oceanobacillus sp. LNSP1E3-1			96.8	
Oceanobacillus sp. LNSP9E3-2.1			96.6	
Oceanobacillus sp. LNSP1E3-1.1			97.7	
Oceanobacillus sp. LNSP9E3-1.2			97.5	
Oceanobacillus sp. LNSP10E3-2_0			98	
Oceanobacillus sp. LNSP3E3-1			97.6	
Oceanobacillus sp. LNSP3E3-2			98	

[a] Similarity percent; ^{T} type strain; NA: Not apply.
