# Supplementary material for: Diversity and Biotechnological Potential of Cultivable Halophilic and Halotolerant Bacteria from the “Los Negritos” Geothermal Area
Source: Microorganisms. 2024 Feb 27;12(3):482. doi: 10.3390/microorganisms12030482 (PMC10972316; doi:10.3390/microorganisms12030482)
Supplement: Supplementary file 1 [file microorganisms-12-00482-s001.zip › Table-S3.pdf]

Table S3. Plant growth promotion characteristics showed by halotolerant and halophilic strains from “Los Negritos” – Villamar – Michoacán state.

Strain	Siderophore		IAA (µg mL^{-1})
	1% NaCl	10% NaCl	
Bacillus sp. LNSP2103-3	1.34	-	-
Bacillus sp. LNSP2103-4	1.42	-	-
Halobacillus sp. LNHM5103-1	-	-	1.82
Halobacillus sp. LNHM4103-1	-	-	1.13
Halomonas sp. LNSP10E3-2.1	1.31	-	-
Halomonas sp. LNSP4E3-1	1.58	+	-
Halomonas sp. LNSP2E3-1	1.16	-	-
Halomonas sp. LNSP6E3-2	1.35	-	-
Halomonas sp. LNSP6-1	2.09	-	-
Halomonas sp. LNSP5E3-1	1.00	-	-
Halomonas sp. LNSP5E3-2	1	-	-
Halomonas sp. LNSP4103-1	1.77	-	-
Halomonas sp. LNSP5E3-1.1	1	-	-
Halomonas sp. LNSP5E3-2.2	1.07	-	-
Halomonas sp. LNSP3103-1	1.19	-	-
Kocuria sp. LNSP5103-1	1.38	-	-
Marinococcus sp. LNHM4E3-1			0.63
Marinococcus sp. LNHM3E3-2.2			0.57
Marinococcus sp. LNHM5E3-2.1			0.63
Nesterenkonia sp. LNSP9103-1	1.80	-	-
Oceanobacillus sp. LNSP10E3-2	2.17	-	-
Oceanobacillus sp. LNSP3E3-1	1.76	+	-
Oceanobacillus sp. LNSP3E3-2	1.33	-	-
Oceanobacillus sp. LNSP8E3-2	1.67	-	-
Priestia sp. LNSP6-2	1.53	-	-
Salibacterium sp. LNHM5E3-1	-	-	7.93
Salibacterium sp. LNHM5E3-2.2	-	-	9.18
Salimicrobium sp. LNHM3E3-1			0.26
Salimicrobium sp. LNHM2E3-1			0.15
Salimicrobium sp. LNHM10E3-1			0.44
Salinicoccus sp. LNSP10E3-1.1	1.56	-	-
Staphylococcus sp. LNSP7E3-1.1	1.30	-	-
Staphylococcus sp. LNSP7E3-2	1.21	+	-
Virgibacillus sp. LNSP2E3-1.1	-	+	-
Virgibacillus sp. LNSP10E3-1	-	-	5.79
